# Irinotecan-Induced Steatohepatitis: Current Insights

**DOI:** 10.3389/fonc.2021.754891

**Published:** 2021-10-11

**Authors:** Jun Han, Jing Zhang, Chengliang Zhang

**Affiliations:** ^1^ Tongji Hospital, Tongji Medical College, Huazhong University of Science and Technology, Wuhan, China; ^2^ Department of Pharmacy, Affiliated Hospital of Jianghan University, Wuhan, China; ^3^ Wuhan Red Cross Hospital, Wuhan, China

**Keywords:** irinotecan, chemotherapy, hepatotoxicity, hepatic steatosis, steatohepatitis

## Abstract

The hepatotoxicity of irinotecan is drawing wide concern nowadays due to the widespread use of this chemotherapeutic against various solid tumors, particularly metastatic colorectal cancer. Irinotecan-induced hepatotoxicity mainly manifests as transaminase increase and steatosis with or without transaminase increase, and is accompanied by vacuolization, and lobular inflammation. Irinotecan-induced steatohepatitis (IIS) increases the risk of morbidity and mortality in patients with colorectal cancer liver metastasis (CRCLM). The major risks and predisposing factors for IIS include high body mass index (BMI) or obesity, diabetes, and high-fat diet. Mitochondrial dysfunction and autophagy impairment may be involved in the pathogenesis of IIS. However, there is currently no effective preventive or therapeutic treatment for this condition. Thus, the precise mechanisms underlying the pathogenesis of IIS should be deciphered for the development of therapeutic drugs. This review summarizes the current knowledge and research progress on IIS.

## Introduction

**Figure 1:**
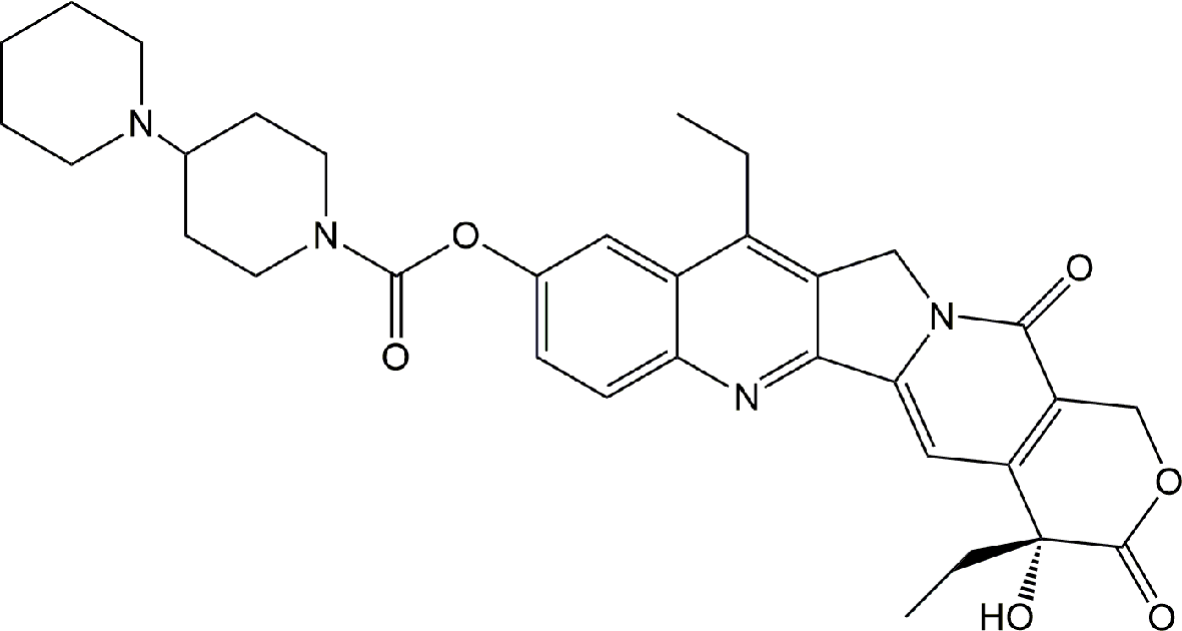
The structure of irinotecan.

Irinotecan, also termed as CPT-11 or 7-ethyl-10-[4-(1-piperidino)-1-piperidino]-carbonyloxycamptothecine (**Figure 1**), is an inhibitor of DNA topoisomerase I and has been used for 27 years since it was first approved in Japan in 1994 (1). As a crucial chemotherapeutic agent, it is widely used either alone or in combination against various solid tumors, particularly for the treatment of metastatic colorectal cancer, as recommended by the guidelines of the National Comprehensive Cancer Network and the European Society for Medical Oncology (1, 2). Notably, irinotecan-based neoadjuvant chemotherapy has improved the five-year survival rate in colorectal cancer liver metastasis (CRCLM) with unresectable tumors by approximately 58% (3, 4). However, there is a growing realization that irinotecan-induced hepatotoxicity, such as hepatic steatosis and steatohepatitis, can increase the risk of morbidity and mortality in patients with CRCLM (5, 6). Although irinotecan-induced steatohepatitis (IIS) has been known to be a clinicopathological symptom of irinotecan for decades, the mechanisms underlying this adverse effect are not exactly known. This review provides current insights into the clinical understanding of the epidemiology, risk factors, possible causal mechanisms, as well as preventive and therapeutic approaches regarding IIS.

## Brief Description of Irinotecan

Irinotecan, which is a semi-synthetic and water-soluble camptothecin-derivative cytotoxic drug (2). It inhibits the DNA-topoisomerase I complex and causes DNA double-strand breaks, thereby inducing cytotoxicity (7, 8). As a prodrug, irinotecan is metabolized to the active metabolite SN-38, also termed as 7-ethyl-10-hydroxycamptothecin, in the blood and liver mainly by human carboxylesterase 2 (1, 9). Compared with irinotecan, SN-38 is a stronger inhibitor of DNA topoisomerase I (10, 11) and can induce lethal DNA double-strand breaks and eventually cell death (9, 11). SN-38 is inactivated upon its conversion into SN-38G (β-glucuronide conjugate) by uridine diphosphate-glucuronosyl transferase 1A1 (UGT1A1) in the liver. SN-38G can be converted back into SN-38 by bacterial β-glucuronidase in the intestinal tract, and the resulting SN-38 is absorbed into the systemic circulation, whereby the anti-tumor effect of irinotecan is extended (9, 12).

Unfortunately, irinotecan can non-specifically damage any rapidly proliferating cell, including both tumor cells and non-tumor cells, such as bone-marrow cells and intestinal basal cells, as well as the commensal bacteria in the body (1). Consequently, hematotoxicity (neutropenia) and gastrointestinal toxicity (diarrhea) are common irinotecan-induced toxicities (8), with a large inter-individual variation (1). In recent years, an increasing body of evidence has demonstrated that irinotecan-induced hepatotoxicity, including hepatic steatosis and steatohepatitis can increase the risk of morbidity and mortality in patients with CRCLM (6). Therefore, IIS is nowadays drawing increasing attention in clinical practice.

## Epidemiology of IIS

Long-term or high-dose administration of irinotecan may impair the liver parenchyma, thus leading to hepatotoxicity with or without transaminase increase (7, 13–16). There are numerous epidemiological reports on IIS, which mainly focus on patients with CRCLM. However, there are significant differences in IIS incidence among these studies. Morris-Stiff et al. summarized that up to 50% of patients with CRCLM who receive neoadjuvant irinotecan develop IIS (17). In a prospective study involving 45 patients with CRCLM who underwent hepatic resection, Gomez-Ramirez et al. observed that four out of the seven patients (57.2%) who had received neoadjuvant irinotecan developed IIS (18). Pawlik and colleagues analyzed 153 patients with CRCLM and reported that moderate or severe hepatic steatosis was dramatically more frequent in the patients who had received neoadjuvant irinotecan (n = 15, 27.3%) than in those without any chemotherapy (n = 2, 3.4%) or with 5-FU (n = 10, 14.9%) or oxaliplatin (n = 3, 9.6%) monotherapy (19). A meta-analysis found that one in every twelve patients with CRCLM under irinotecan-based regimens will ultimately develop IIS (20). By analyzing 406 patients with CRCLM who had undergone hepatic resection, Vauthey and co-workers showed that irinotecan is related to IIS (20.2% vs. 4.4% of the patients without chemotherapy) (21). Moreover, although liver biopsy, which is the gold standard in diagnosing steatosis or steatohepatitis (6), is recommended to diagnose IIS (22), sampling error and observational variations among pathologists can affect the diagnosis (23).

IIS is associated with the disruption of lipid homeostasis and with inflammation in hepatic cells. It may progressively increase the risk of fibrosis, cirrhosis and liver failure (24, 25), because irinotecan-based regimens have potentially harmful effects on liver parenchyma and associated with impaired liver regeneration (17, 26). Vauthey et al. found that IIS remarkably increased the 90-day mortality of patients with CRCLM (14.7% vs. 1.6% of those with no IIS) (21). The presence of IIS is more concerning than simple steatosis when undergoing major liver resection and has been demonstrated to be associated with increased surgical morbidity and mortality after resection of colorectal liver metastases. Morris-Stiff and colleagues found that IIS is related to increased morbidity and possibly to increased mortality in patients with CRCLM following hepatectomy because of the development of liver failure (17). The detrimental effect of hepatic steatosis in patients undergoing liver resection was also demonstrated in a meta-analysis by Robinson et al. (20). Therefore, it is crucial to emphasize that careful consideration needs to be given when performing extensive procedures on patients with IIS.

## Risk Factors for IIS

Multiple studies have found that confounding factors could impact the development of chemotherapy-associated steatohepatitis (CASH) (including IIS) (26–28). High body mass index (BMI) or obesity is closely related to an increased risk of CASH (14, 29). Patients with BMI ≥ 25 kg/m^2^ under irinotecan-based chemotherapy have a 2.03-fold risk of IIS compared with those with BMI < 25 kg/m^2^ (21). Another report by Ryan et al. noted that both IIS and hepatic steatosis are correlated with a BMI of ≥ 30 kg/m^2^ (30). In a small cohort study, patients with a high BMI who had undergone irinotecan-based chemotherapy were found to be associated with a high IIS score according to the Brunt System (29). Fernandez et al. demonstrated that severe IIS is related to neoadjuvant irinotecan in patients, particularly obese patients, with CRCLM who had undergone hepatic resection (22). Animal experiments have shown that the decreased hepatic UGT1A1 and increased fecal β-glucuronidase levels in diet-induced obese mice compared with the levels in lean mice are responsible for the prolonged retention of SN-38, consequently increasing the occurrence of IIS, in these obese mice (31).

Diabetes may be another important risk factor for IIS. Wolf et al. demonstrated that hepatic steatosis or IIS is more common in diabetic patients treated with irinotecan-based regimens as neoadjuvant chemotherapy before hepatic resection of CRCLM (27). Moreover, a diet with high-fat content may also accelerate the development of IIS. A dietary study by Mallick et al. demonstrated that, upon irinotecan treatment, mice on a high-fat diet, such as lard, develop steatosis more easily than those on a regular diet (32). Although these reports are based on retrospective analyses or animal studies, the results indicate that, upon irinotecan-based chemotherapy, patients on a high-fat diet or with baseline obesity or diabetes may have a higher risk of developing IIS than non-diabetic and non-obese patients on a regular diet. However, this possibility should be verified *via* prospective controlled trials.

## Mechanisms Underlying IIS

**Figure 2:**
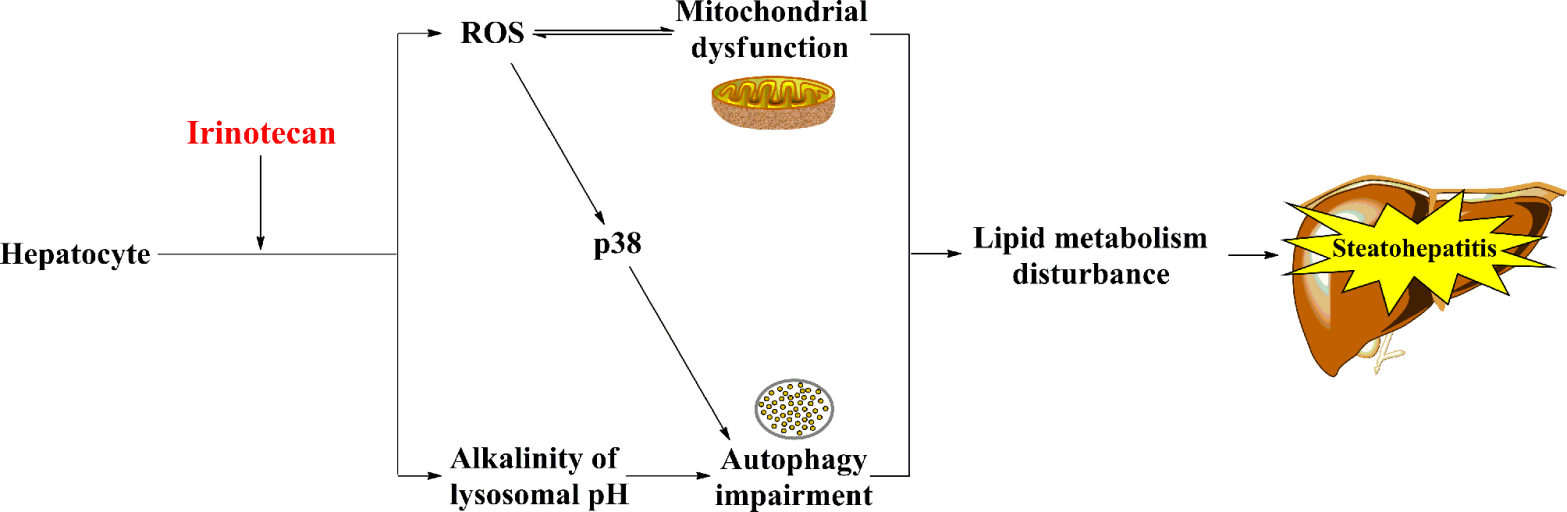
The potential mechanisms of IIS. Irinotecan-caused mitochondrial dysfunction and autophagy impairment result in lipid metabolism disturbance ultimately, which may involve in the pathogenesis of IIS.

The exact mechanisms underlying IIS have not been fully elucidated. However, mitochondrial dysfunction and autophagy impairment have been proposed to be involved in the pathogenesis of IIS (**Figure 2**).

### Mitochondrial Dysfunction

Hepatocytes are rich in mitochondria, which are vulnerable to chemotherapeutic agents (33–35). In general, inhibition of β-oxidation of fatty acids, oxidative phosphorylation, and mitochondrial respiration primarily contributes to mitochondrial dysfunction (7). Irinotecan causes accumulation of lipids in hepatocytes *via* inhibiting the β-oxidation of fatty acids, which is one of the main pathways of the lipid metabolism, in the mitochondria of hepatocytes (7, 25). Moreover, irinotecan induces oxidative stress by uncoupling oxidative phosphorylation, restraining mitochondrial respiration, and facilitating the mitochondrial release of reactive oxygen species (ROS) in hepatocytes (7, 36). Interestingly, Bao et al. demonstrated that SN-38 upregulated ROS in cells derived from primary human hepatocytes but not in cancer cells (MDA-MB-231 and T47D) *in vitro* (8). Upregulation of ROS causes mitochondrial dysfunction (37) and stimulates the pathogenesis of IIS (14). It is worth noting that mitochondrial dysfunction usually improves when the chemotherapy is terminated (25).

### Autophagy Impairment

Autophagy is a lysosome-mediated intracellular protein-degradation mechanism. It also regulates the lipid metabolism by metabolizing intracellular lipid droplets (triglycerides), which are the main form of lipid storage in the cell (38). Thus, impairment of this mechanism causes multiple metabolic diseases, such as obesity and hepatic steatosis (39). Mahli et al. found that irinotecan can weaken the autophagic flux by increasing the lysosomal pH to alkalinity, thereby contributing to lipid accumulation and steatosis in primary human hepatocytes (40). Furthermore, irinotecan impairs mitochondrial function and indirectly activates p38, thus inhibiting autophagosome formation (41, 42).

## Potential Preventive and Therapeutic Approaches Against IIS

In general, there is still a lack of effective preventive and therapeutic strategies against IIS due to its complex mechanism of pathogenesis. However, several preclinical studies for IIS have suggested potential interventional drugs or measures, as described below.

### Silymarin

Silymarin is a hepatoprotective agent, and it is derived from the seeds of *Silybum marianum* (43). As a natural flavonoid, silymarin has antioxidative effects and can decrease the oxidative stress in the liver (44). A study by Marcolino et al. about the effect of silymarin on IIS in mice reported that silymarin has a dual effect; low-dosage (1.5 mg/kg) of silymarin prevents irinotecan-induced hepatic injury, such as steatosis, vacuolization, lobular inflammation, and fibrosis, by suppressing the inflammatory factors and oxidative stress in the liver, whereas high-dosage (150 mg/kg) of silymarin exacerbates IIS and increases the mortality (45). Thus, the mechanisms whereby silymarin at different dosages result in different effects remain to be explored, and the specific clinical effects need to be confirmed, in future studies.

### Pioglitazone

Pioglitazone is a thiazolidinedione antidiabetic agent. It modulates the lipid metabolism and ameliorates the glycemic control in patients with type-2 diabetes *via* activating the peroxisome proliferator-activated receptor (46, 47). A study in rats demonstrated that pioglitazone has a hepatoprotective effect against chemotherapy (irinotecan and 5-fluorouracil)-induced steatohepatitis, but no effect on histopathological changes (24). Therefore, the hepatoprotective effect of pioglitazone against IIS should be further explored.

### Sorafenib

Sorafenib, a multityrosine-kinase inhibitor, is used for the treatment of unresectable hepatocellular carcinoma and advanced renal cell carcinoma (48). Mahli and co-workers reported that sorafenib has a protective effect against IIS by decreasing irinotecan-induced ERK activation and pro-inflammatory gene expression in hepatocytes and murine models of IIS (40). Nevertheless, the hepatoprotective effect of sorafenib against IIS should be confirmed in patients *via* clinical studies.

### Glycine

Glycine is a nonessential amino acid with remarkable protective effects against liver injury (49, 50). Mikalauskas et al. found that glycine markedly reduces the levels of transaminases and microvesicular steatosis in rats treated with FOLFIRI, presumably by inhibiting the activation of Kupffer cells and enhancing the hepatic microcirculation (51).

### Grain-Based Chow Diet

Phytoestrogens (especially isoflavones) and polyunsaturated fatty acids (PUFA) in diet may be effective in suppressing non-alcoholic fatty liver disease (NAFLD) or IIS. Isoflavones can be beneficial against NAFLD by reducing the lipogenesis, lipolysis, and fat deposition in adipocytes (52, 53). PUFA can decrease hepatic storage of triglycerides and has a significant protective effect against hepatic steatosis (54). A dietary study by Mallick et al. reported that a grain-based chow diet, which included a low level of fat (vegetable-based, such as soybean oil, which is especially rich in PUFA) and high levels of carbohydrate (fiber), phytoestrogen, and protein, had a notably protective effect against irinotecan-induced mixed hepatic steatosis (micro & macrovesicular) in mice (32). Thus, similar dietary studies involving specific ingredients should be performed on cancer patients undergoing irinotecan treatment.

## Conclusions

Overall, IIS is a crucial adverse effect of irinotecan and can increase the risk of morbidity and mortality in patients with CRCLM. The major risks and predisposing factors for IIS include high BMI or obesity, diabetes, and high-fat diet. Although mitochondrial dysfunction and autophagy impairment may be involved in the pathogenesis of IIS, the exact causal mechanisms of IIS have not been fully elucidated. Till now, liver biopsy is the gold standard in diagnosing hepatotoxicity, including IIS, but it is a highly invasive, complex, and painful operation. Thus, biomarkers of IIS, are urgently needed to precisely evaluate irinotecan-induced hepatotoxicity. Besides, we should explore more risk factors for the development of IIS which can help oncologists to identify the patients at risk. Furthermore, effective preventive and therapeutic approaches are still lacking. Potential interventional drugs or measures have been reported in multiple preclinical studies and the medications susceptible to be active in steatosis such as fibroblast growth factor 21 agonists, obeticholic acid or glucagon-like peptide-1 agonists deserve considerations. Thus, further investigations involving humans, especially clinical trials, are required to develop feasible preventive and therapeutic approaches against IIS.

## Author Contributions

CZ designed this work. JH and JZ wrote this manuscript. All authors contributed to the article and approved the submitted version.

## Funding

This work was supported by grants from the scientific research project of Wuhan NO.6 hospital (No. LX19013 to JH).

## Conflict of Interest

The authors declare that the research was conducted in the absence of any commercial or financial relationships that could be construed as a potential conflict of interest.

## Publisher’s Note

All claims expressed in this article are solely those of the authors and do not necessarily represent those of their affiliated organizations, or those of the publisher, the editors and the reviewers. Any product that may be evaluated in this article, or claim that may be made by its manufacturer, is not guaranteed or endorsed by the publisher.

## Abbreviations

BMI, body mass index; CASH, chemotherapy-associated steatohepatitis; CRCLM, colorectal cancer liver metastasis; IIS, irinotecan-induced steatohepatitis; NAFLD, non-alcoholic fatty liver disease; PUFA, polyunsaturated fatty acids; ROS, reactive oxygen species; UGT1A1, uridine diphosphate-glucuronosyl transferase 1A1.
